# Prelabor rupture of membranes at term: A possible hematological triage in addition to vagino-rectal beta-hemolytic streptococcus screening for early labor induction

**DOI:** 10.1371/journal.pone.0261906

**Published:** 2022-01-13

**Authors:** Francesco D’Ambrosi, Nicola Cesano, Enrico Iurlaro, Alice Ronchi, Ilaria Giuditta Ramezzana, Matteo Di Maso, Carlo Pietrasanta, Andrea Ronchi, Lorenza Pugni, Enrico Ferrazzi

**Affiliations:** 1 Department of Woman Child and Neonate, Obstertics Unit, Fondazione IRCCS Ca’ Granda Ospedale Maggiore Policlinico, Milan, Italy; 2 Department of Clinical Sciences and Community Health, Branch of Medical Statistics, Biometry and Epidemiology “G.A. Maccacaro”, University of Milan, Milan, Italy; 3 Unit of Neonatal Intensive Care, Fondazione IRCCS Ca’ Granda Ospedale Maggiore Policlinico, Milan, Italy; 4 Department of Clinical Sciences and Community Health, University of Milan, Milan, Italy; University of Washington, UNITED STATES

## Abstract

**Introduction:**

A potential complication of term prelabor rupture of membranes (term PROM) is chorioamnionitis with an increased burden on neonatal outcomes of chronic lung disease and cerebral palsy. The purpose of the study was to analyze the efficacy of a standing clinical protocol designed to identify women with term PROM at low risk for chorioamnionitis, who may benefit from expectant management, and those at a higher risk for chorioamnionitis, who may benefit from early induction.

**Material and methods:**

This retrospective study enrolled all consecutive singleton pregnant women with term PROM. Subjects included women with at least one of the following factors: white blood cell count ≥ 15×100/μL, C-reactive protein ≥ 1.5 mg/dL, or positive vaginal swab for beta-hemolytic streptococcus. These women comprised the high risk (HR) group and underwent immediate induction of labor by the administration of intravaginal dinoprostone. Women with none of the above factors and those with a low risk for chorioamnionitis waited for up to 24 hours for spontaneous onset of labor and comprised the low-risk (LR) group.

**Results:**

Of the 884 consecutive patients recruited, 65 fulfilled the criteria for HR chorioamnionitis and underwent immediate induction, while 819 were admitted for expectant management. Chorioamnionitis and Cesarean section rates were not significantly different between the HR and LR groups. However, the prevalence of maternal fever (7.7% vs. 2.9%; p = 0.04) and meconium-stained amniotic fluid was significantly higher in the HR group than in LR group (6.1% vs. 2.2%; p = 0.04). This study found an overall incidence of 4.2% for chorioamnionitis, 10.9% for Cesarean section, 0.5% for umbilical artery blood pH < 7.10, and 1.9% for admission to the neonatal intensive care unit. Furthermore, no confirmed cases of neonatal sepsis were encountered.

**Conclusions:**

A clinical protocol designed to manage, by immediate induction, only those women with term PROM who presented with High Risk factors for infection/inflammation achieved similar maternal and perinatal outcomes between such women and women without any risks who received expectant management. This reduced the need for universal induction of term PROM patients, thereby reducing the incidence of maternal and fetal complications without increasing the rate of Cesarean sections.

## Introduction

Term prelabor rupture of membranes (term PROM) is defined as the rupture of fetal membranes before the onset of labor at a gestational age > 37 weeks [1]. The global incidence of term PROM is approximately 8% [1]. Spontaneous onset of labor follows PROM within 12 hours in 79% of the patients and within 24 hours in 95% of the patients [2].

Rare, immediate risks associated with term PROM include cord prolapse or compression and/or placental abruption [3–6]. A relatively more frequent and potential complication is chorioamnionitis with an increased burden on neonatal outcome(s) including sepsis, the respiratory distress syndrome, and brain injury [7–11].

In term pregnancy, the incidence of chorioamniositis varies from 6 to 10% and occurs in 40% of prolonged PROM that persists for more than 24 hours [7]. Other risk factors described are prolonged labor, multiple vaginal exams, nulliparity, Afro-American ethnicity, internal monitoring of labor, meconium-stained amniotic fluid, alcohol or drug abuse, smoking, immune-compromised states, epidural anesthesia, vaginal colonization with group B streptococcus, bacterial vaginosis, sexually transmissible genital infections and vaginal colonization with ureaplasma [5].

The passage of infectious organisms to the chorio-amnioniotic membranes or umbilical cord of the placenta is the first step in the pathogenesis of chorioamnionitis.

The infectious agents can arrive by retrograde or ascending infection from the lower genital tract from vagina and cervix, or via hematogenous transmission. A combination of proinflammatory and inhibitory cytokines and chemokines are released in the maternal and fetal compartments as response to the infection. The inflammatory response and the production of prostaglandin may cause clinical chorioamnionitis with ripening of the cervix, membrane injury, and labor at term or premature birth. The fetus presents a direct risk of infection, or of inflammatory dysfunction mainly of the central nervous system and lungs which may result in short and long-term neurologic deficits and respiratory distress [5].

A planned early induction of labor after term PROM may prevent such complication(s) at the cost of an increased risk of delivery by Cesarean section (CS) [4, 9].

The purpose of the present study was to assess whether routine low-cost hematological parameters of infection and inflammation and early induction of only positive cases at hematological triage may benefit both, high-risk women (who are to be induced promptly) and low-risk women (who are admitted for expectant management).

## Material and methods

This retrospective study we have considered all consecutive women with singleton pregnancy who were referred to the authors’ obstetrics unit at the *Fondazione IRCCS Ca’ Granda Ospedale Maggiore Policlinico*, *Mangiagalli* Maternity Centre for term PROM. Suspected term PROM at referral and objective examination was confirmed by a positive test for placental alpha microglobulin-1 protein in the vaginal fluid. In our country in the presence of PROM, hospitalization is always required [10].

All consecutive patients with a fetus in the cephalic presentation and an onset of term PROM <4 hour before referral to the hospital were included in this study. Women with pre-existing diabetes, gestational diabetes, hypertensive disorders of pregnancy, autoimmune disorders or cholestasis, history of uterine surgery, maternal fever ≥38°C, non-reactive fetal heart rate monitoring or persistent fetal tachycardia, uncertain dating of pregnancy, multiple pregnancies, breech presentation, abnormal placenta insertion, planned CS, and known fetal malformations were excluded from the study.

At the time of admission, all patients underwent venous blood sampling for white blood cell (WBC) and C-reactive protein (CRP) assessment. Patients presenting with at least one of the following factors—WBC count ≥15×100/μL, CRP ≥1.5 mg/dL, and/or positive vaginal swab for beta-hemolytic streptococcus in the previous 4 weeks—were allocated to the high-risk (HR) group and underwent immediate induction of labor by intravaginal dinoprostone.

In fact, according to exiling national guidelines all women undergo a screening swab which must be performed within 4 weeks before giving birth [10].

Women presenting with none of the above factors were allocated to the low-risk (LR) group and monitored for up to 24 hours for spontaneous onset of labor; after 24 hours, patients in this group were induced by intravaginal prostaglandins. Antibiotic prophylaxis was administered at the time of diagnosis of PROM in HR group and after 12 hours from rupture of membranes tin the LR groups, respectively (in accordance with the local protocol) [11]. Intravenous prophylaxis was the same for both groups: 2 g of ampicillin, followed by 1 g every 4 hours until delivery [11, 12].

The primary outcome of the study was the incidence of maternal chorioamnionitis, defined as the presentation of maternal fever (≥38°C) and at least two of the following: maternal leukocytosis (≥15×100/μL in absence of corticosteroids, fetal persistent mild tachycardia (>160 beats/min) or purulent fluid from the cervical os (cloudy or yellowish thick discharge confirmed visually on speculum exam to be coming from the cervical canal) [8].

Secondary outcomes were the CS rate, umbilical arterial blood pH < 7.10, Apgar score < 7 at 5 min, and neonatal outcomes including short-term postnatal resuscitation maneuvers on admission, late admission to the neonatal intensive care unit, sepsis, assisted ventilation, and antibiotic therapy. Sepsis was confirmed by the presence of clinical signs and symptoms suggestive of infection and a positive blood culture for bacteria [11, 12]. Sepsis was defined as “suspected” when the patient presented with clinical signs or symptoms suggestive of infection, but a negative blood culture for bacteria, but the requirement for intravenous antibiotic therapy for at least 5 days in accordance with the clinician’s judgment [13–15].

In particular, in the presence of clinical signs suggestive of early neontal sepsis (sepsis which occurs in the first 72 hours of life) diagnostic tests are performed (blood count, CRP, blood culture and possibly liquorculture) and empiric antibiotic therapy with ampicillin and gentamicin is undertaken, with the addition of a third generation cephalosporin in case of possible involvement of the central nervous system [13–16].

This retrospective study (PROM) was approved by the Institutional Review Board of Fondazione IRCCS Ca’ Granda, Ospedale Maggiore Policlinico, Milan, Italy (September 17, 2020, PROM0115032021). Each patient provided informed written consent for the use of anonymized clinical records for non-pharmacological research purposes at admission.

### Statistical analysis

Data was analysed using the Statistical Package for Social Science (SPSS, version 23.0, IL, USA). Categorical variables are expressed as absolute frequencies (number of women per group) and percentages, whereas continuous variables are expressed as means and standard deviations (SD). Differences in the means and proportions between the LR and HR groups were evaluated using the Student’s *t* test and the chi-squared test, respectively. The primary outcome was the composite rate of adverse chorioamnionitis. The 95%CI of the proportion was calculated using a binomial distribution model. The sample size (about 800 women) was calculated expecting a 5% rate of chorioamnionitis [17] and aiming at providing an estimation of this rate with a precision of ± 1.5% (i.e., with an overall 95%CI of the observed proportion of about 3%). This level of precision was arbitrarily decided by the authors.

## Results

From January 1, 2018 to June 30, 2019, a total of 928 consecutive pregnant women diagnosed with PROM were retrospectively analyzed for this study. Of these, 44 were excluded because they did not fulfill the inclusion criteria: pre-existing pregnancy complications (n = 35), multiple pregnancies (n = 5), fetal malformations (n = 2), and breech presentations (n = 2).

In 884 subjects, out of 928, who met the inclusion criteria, 65 (7%) were in the HR group with 819 (93%) subjects remaining in the LR group. The baseline characteristics of the patients allocated to the HR and LR groups, are reported in Table 1. Of the 65 patients in the HR group, 30 (46.2%) had only a positive vaginal swab for beta-hemolytic streptococcus, 18 (27.7%) CRP ≥1.5 mg/dL, 11 (16.9%) had only WBC count ≥15×100/μL, 3 (4.6%) presented elevated WBC and CPR, in 2 (3.1%) cases positive vaginal swab was associated with elevate WBC and in 1 (1.5%) with elevated CPR.

**Table 1:** Maternal and obstetric characteristics of 819 women with negative inflammatory indices and negative vaginal swab for beta-hemolytic streptococcus (LR-group) and 65 with positive inflammatory indices and/or positive vaginal swab for beta-hemolytic streptococcus (HR-group). (Data are reported as number of cases and percentage in brackets, or mean and standard deviation, where appropriate).

	Inflammatory indices		p-value
	Negative Expectant management	Positive early induction	
	(n = 819)	(n = 65)	
**Maternal demographic**			
Maternal Age (year)	33.4 (5.0)	33.1 (5.3)	p = 0.93
Multiparous women	223 (27.2)	16 (24.6)	p = 0.65
South European Caucasian ancestry	715 (87.3)	59 (90.7)	p = 0.42
**Induction and labour**			
Bishop score (*)	3.3 (0.9)	3.3 (0.9)	p = 0.87
Induced labour	188 (22.9)	65 (100)	N.A.
Analgesia	591 (72.2)	53(81.5)	p = 0.10
**Maternal fever during labour**	**24 (2.9)**	**5 (7.7)**	**p = 0.04**
Fetal tachycardia	17 (2.1)	2 (3.2)	p = 0.59
**Meconium-stained amniotic fluid**	**18 (2.2)**	**4 (6.1)**	**p = 0.05**
Chorioamnionitis	32 (3.9)	5 (7.6)	p = 0.14
**Interval from PROM to delivery (hours)**	**23.1 (16.0)**	**28.5 (11.6)**	**p = 0.02**
Mode of delivery			p = 0.80
Vaginal delivery	650 (79.3)	52 (80.0)	
Operative vaginal delivery	81 (9.8)	5 (7.6)	
Caesarean Section	88 (10.6)	8 (12.3)	
Indications for operative delivery			p = 0.05
Maternal Fever	1 (0.1)	1 (1.5)	
Failed induction	6 (0.7)	2 (3.0)	
Fetal distress (**)	64 (7.8)	7 (10.7)	
Failure to progress 1st stage	17 (2.0)	0 (0.0)	
Failure to progress 2nd stage	79 (9.6)	3 (4.6)	
Placental abruptio	2 (0.2)	0(0.0)	

* Calculated on 188 patients induced, after 24 hours, in the LR-group.

** Defined as persistent 2nd or 3rd Group of fetal heart rate tracing according to ACOG classification [18].

Labor began spontaneously within 24 hours in 631 (77.0%) women in the LR group. The incidence of chorioamnionitis (3.9% vs 7.6%; p = 0.14) and CS (10.6% vs 12.3%; p = 0.80) did not differ significantly between the LR and HR groups. Among the 30 women induced only due to positive swabs for beta-hemolytic streptococcus (in the HR group), we observed two cases (6.7%) of chorioamnionitis. The prevalence of maternal fever (p < 0.04) and meconium-stained amniotic fluid (p < 0.05) were significantly higher in the HR group respectively 7.7% and 6.1% than in the LR group, 2.9% and 2.2%. The LR group exhibited a significantly shorter PROM-to-delivery time (expressed in hours) as compared to the HR group (p < 0.02). No other significant differences in the maternal outcome during labor and postpartum were observed between the two groups (Table 1).

The maternal and neonatal outcomes are presented in Table 2. No clinical index of maternal or neonatal outcome was significantly different between the two groups. No case had an Apgar score < 7 in this study.

**Table 2:** Maternal and neonatal outcome of 819 women with negative inflammatory indices (LR-group) and 65 with positive (HR-group) and/or positive vaginal swab for beta-hemolytic streptococcus. (data are reported as number of cases and percentage in brackets, or mean and standard deviation, where appropriate).

	Inflammatory indices		p-value
	Negative Expectant management	Positive early induction	
	(n = 819)	(n = 65)	
**Maternal outcome**			
Perineal tears 3rd– 4th degree	3 (0.4)	0 (0.0)	p = 0.63
Blood transfusion	9 (1.1)	2 (3.0)	p = 0.17
Hysterectomy	1 (0.1)	0 (0.0)	p = 0.78
Days of maternal hospitalization	4.2 (2.0)	4.3 (1.5)	p = 0.53
**Neonatal outcome**			
Gestational age (weeks)	38.9 (1.3)	39.2 (1.2)	p = 0.30
Birth weight (g)	3226 (381.0)	3319 (398.6)	p = 0.67
Apgar score at 1 min <5	7 (0.9)	0 (0.0)	p = 0.45
Umbilical artery pH<7.10	4 (0.5)	0 (0.0)	p = 0.57
Primary Resuscitation in delivery room (*)	7 (0.9)	0 (0.0)	p = 0.13
Admission to NICU	15 (1.8)	2 (3.0)	p = 0.48
Back admission to NICU from rooming-in	17 (2.0)	3 (4.6)	p = 0.19
Suspected sepsis	12 (1.5)	1 (1.5)	p = 0.96
Proven Sepsis	0 (0.0)	0 (0.0)	n.p.
Antibiotics therapy	18 (2.2)	3 (4.6)	p = 0.22
Assisted ventilation (**)	9 (1.0)	0 (0.0)	p = 0.58

* by facial mask

** One case of mechanical ventilation and eight cases of non-invasive respiratory support.

The overall outcomes of the entire cohort are summarized in Table 3: we detected in our population a prevalence of chorioamnionitis of 4.2%, with a CS rate of 10.9%.

**Table 3:** Outcome and incidence of complication in our population (data are reported as number of cases and percentage in brackets).

Outcome	Population
	n = 884
**Maternal Outcome**	
*Caesarean sections*	96 (10.9)
*Chorioamnionitis*	37 (4.2)
*Perineal tears 3*^*rd*^*– 4*^*th*^ *degree*	3 (0.3)
*Vaginal haematoma*	2 (0.2)
*Post partum haemorrhage > 500 cc*	230 (26.0)
*Blood transfusion*	11(1.2)
*Placental retention*	2 (0.2)
*Hysterectomy*	1 (0.1)
**Neonatal Outcome**	
*Neonatal resuscitation manoeuvres*	7 (0.8)
*Admission to NICU*	17 (1.9)
*Umbilical artery pH <7*.*10*	4 (0.5)
*Suspected sepsis*	13 (1.5)
*Sepsis*	0 (0.0)
*Mechanic ventilation*	1 (0.1)
*Antibiotics therapy*	21 (2.4)

Among the primary outcomes observed in this cohort, the major maternal or neonatal complications did not have a significant prevalence in the expectant management group. We observed one case of postpartum hysterectomy for severe postpartum hemorrhage and four (0.5%) cases of respiratory acidosis at birth. Regarding neonatal outcomes, seven (0.9%) newborns required resuscitation maneuvers immediately after birth. Furthermore, sepsis was suspected in 13 (1.5%) cases; however, it was ruled out eventually by blood culture analysis. Antibiotic therapy was adopted in only 21 cases (2.4%) (Table 3).

## Discussion

Our study demonstrated how identifying a high-risk population for infections using hematological triage in addition to vagino-rectal beta-hemolytic streptococcus screening can reduce complications such as chorioamnionitis incidence and adverse perinatal outcomes without increasing the number of CS.

The overall prevalence of chorioamnionitis in this retrospective cohort of women with term PROM was 4.2%, with a CS rate of 10.9%. As expected, we found higher incidences of both maternal fever during labor and meconium-stained fluid among women in the HR group presenting with positive inflammatory indices and/or vagino-rectal positive swabs for beta-hemolytic streptococcus. However, it is noteworthy that the difference in the incidence of chorioamnionitis between the subgroup with positive inflammatory indices (7.6%) and the subgroup with negative inflammatory indices (3.9%) was not significantly different (p = 0.14).

The incidence of chorioamnionitis and adverse maternal and perinatal outcomes were similar between the two groups. This was the result of: 1) permitting patients with term PROM and with normal indices of infection to wait up to 24 hours for entering spontaneous labor as their best possible management option and 2) inducing labor in only a minor subgroup of term PROM patients with a biological risk of developing chorioamnionitis, either due to hematological indices of infection or vaginal contamination with beta-hemolytic streptococcus.

In 2017, a Cochrane review [17] reported a comparison between early induction of labor and expectant management in women with term PROM. Methods of induction included intravenous oxytocin, vaginal or oral prostaglandin E2, sublingual or vaginal misoprostol, caulophyllum, and acupuncture. No clear differences between the groups were observed for postpartum pyrexia, postpartum antibiotic administration, rate of CS for fetal distress, rate of operative vaginal births, uterine rupture, epidural analgesia, postpartum hemorrhage, cord prolapse, stillbirth, neonatal mortality, pneumonia, Apgar score < 7 at 5 min, use of mechanical ventilation, and abnormalities in the neonatal cerebral ultrasound.

According to this systematic review, women who underwent early induction demonstrated a significantly lower risk for maternal infection (chorioamnionitis and/or endometritis) than those who underwent expectant management (5.4% vs. 11%). However, no significant differences in the rates of CS were observed between women undergoing induction and those receiving expectant management; the incidence of CS was 12.6% in the induction group and 15% in the expectant management group. Neonatal sepsis occurred in 2.2% of the expectant women with term PROM and in 1.2% of the women who received labor induction. Neonatal sepsis was suspected in 4.1% of the expectant women with term PROM and in 3% of the women in the induced group. The incidence of perinatal mortality was 0.2% in the expectant group and 0.1% in the induced group. According to the authors of this systematic review, the quality of the evidence in the 23 trials analyzed, which included 8,615 cases, was either “low” or “very low”.

Contradictory results are found even among single, well-designed studies addressing the balance between the reduction of the risk for chorioamnionitis and neonatal complications, without a concomitant increase in the rate of CS in women with term PROM undergoing immediate induction of labor as compared to those receiving expectant management [9, 17, 18–20].

In 1996, a seminal study investigating term PROM by Hannah et al. [9] analyzed 5,041 women with PROM. Patients were randomly assigned to four groups: two groups undergoing immediate induction of labor (one with intravenous oxytocin and the other with vaginal prostaglandin E2 gel) and two control groups receiving expectant management for up to 4 days after the onset of term PROM. Clinical chorioamnionitis was detected in 4% of the women immediately induced with oxytocin and in 6.2% of the women induced with prostaglandins. Furthermore, among the two control groups, the prevalence of chorioamnionitis was 8.8% in one group and 7.8% in the other group. The rate of CS in the four groups ranged from 9.6% in the prostaglandin-induced group to 10.9% in the expectant control group. Cord blood pH < 7.10 was observed in 1.4–2.3% of the subjects. Antibiotics were administered to 12.5%, 7.5%, and 10.9% of the newborns in the expectant, oxytocin-induced, and prostaglandin-induced groups. Ventilation after initial resuscitation was necessary in 0.6–1% of the total newborns.

da Graça Krupa et al. [19] evaluated the maternal and perinatal outcomes in patients with term PROM undergoing immediate induction of labor with vaginal misoprostol or receiving expectant management for 24 hours followed by induction. The rate of maternal infection was not reported; however, the rate of CS was 30.7% in the expectant group and 20% in the misoprostol group. There were no differences between the two groups regarding fetal well-being, complications during labor and delivery, and neonatal or postpartum maternal morbidity.

A similar study by Maqbool et al. [20] analyzed 560 cases of term PROM and compared outcomes of immediate induction using misoprostol and expectant management: 33% and 61% of the women in the induction group and the expectant group delivered by CS, respectively. The rate of chorioamnionitis was 5% in the induction group and 25% in the expectant group. The high rate of CS observed in these latter cohorts probably reflects the clinical background of the centers where the studies were performed—Brazil and Pakistan.

In our study, we stratified the population of women with term PROM to identify those at a higher risk of infection according to the hematological indices of infection/inflammation and/or vagino-rectal swabs positive for beta-hemolytic streptococcus. This enabled us to reduce the number of early inductions without a experiencing a significant increase in the incidence of chorioamnionitis, CS, and/or neonatal complications.

Although there were no significant differences in the outcomes between the HR and LR groups, we believe that an overall incidence of 4.2% for chorioamnionitis and a CS rate as low as 10.9% may be considered a good outcome as compared to the previously reported data. In fact, the incidences of chorioamnionitis, CS, and/or neonatal complication(s) in our study are better as compared to in previous studies in which early induction at the time of membrane rupture was performed routinely.

Furthermore, the choice of selective induction of labor, offered solely to higher-risk populations, may enable iatrogenic intervention, thereby empowering the physiology of labor even in cases with term PROM. This important aspect is highlighted in our cohort by the shorter time-to-delivery observed in pregnant women from the LR group, in whom labor started spontaneously in the vast majority of cases (76.7%) and lasted 5 hours lesser than in women from the HR group who underwent early iatrogenic induction.

Strengths of the present study include the use of a protocol that was strictly applied by all members of the staff as part of the real-world clinical practice of our unit, and as such, was not biased by possible prospective trial-positive effects [21] or the enrollment of each consecutive patient with PROM. According to the design of the study we did non randomized patients in the HR group, this would require far larger number of cases, but we also doubted that a formal information in case of positive signs of inflammation could meet a positive acceptance of an expectant management from pregnant patients at term. Potential additional limitations of this study include: its single-center design, which may have been biased by local attitudes and skills, beta-hemolytic streptococcus swabs were performed per protocol two to four weeks prior to delivery, however the prevalence of GBS positive cases was so small that If any case added in the lag time between swab and delivery, such small number should not change the balance between the two groups. Finally, the external validation is limited by the fact that the enrolled patients were mainly of South European Caucasian ancestry. In addition, we missed an important information since we did not measure maternal satisfaction with regard to the labor process and delivery in the two groups.

## Conclusions

Although term PROM is a frequent event in daily obstetrical practice, few well-designed studies are available that provide a scientific background on term PROM management. Moreover, most current guidelines are still based on the Term Prelabor Rupture of the Membranes (Term PROM) study [9] published in 1996, or on the clinical consensus of experts [22]. The criteria adopted as per the protocol in our center, i.e., to administer early induction only to term PROM patients with hematological evidence of infection/inflammation and with swabs positive for rectovaginal beta-hemolytic streptococcus, enabled us to achieve similar maternal and perinatal results between the subgroup of 65 women at high-risk and the 816 women who did not present with any risks and were managed using the expectant protocol for 24 hours. Similar results were observed in terms of the CS rate, chorioamnionitis incidence, and perinatal outcomes.

## Supporting information

S1 Dataset (XLS)

## References

[1] Prelabor Rupture of Membranes: ACOG Practice Bulletin, Number 217. Obstet Gynecol. 2020 Mar;135:e80–e97. doi: 10.1097/AOG.0000000000003700 32080050

[2] Dum P. Premature rupture of the membranes in term patients: induction of labor versus expectant management. Clinical Obstetrics and Gynecology 1998;41:883–91. doi: 10.1097/00003081-199812000-00012 9917943

[3] Romero R, Gomez-Lopez N, Winters AD, Jung E, Shaman M, Bieda J, et al. Evidence that intra-amniotic infections are often the result of an ascending invasion—a molecular microbiological study. J Perinat Med. 2019 Nov 26;47:915–931. doi: 10.1515/jpm-2019-0297 31693497 PMC7147941

[4] Davey MA, King J. Caesarean section following induction of labour in uncomplicated first births- a population-based cross-sectional analysis of 42,950 births. BMC Pregnancy Childbirth. 2016 Apr 27;16:92. doi: 10.1186/s12884-016-0869-0 27121614 PMC4848820

[5] Tita AT, Andrews WW. Diagnosis and management of clinical chorioamnionitis. Clin Perinatol. 2010 Jun;37:339–54. doi: 10.1016/j.clp.2010.02.003 20569811 PMC3008318

[6] Kim CJ, Romero R, Chaemsaithong P, Chaiyasit N, Yoon BH, Kim YM. Acute chorioamnionitis and funisitis: definition, pathologic features, and clinical significance. Am J Obstet Gynecol. 2015 Oct;213:S29–52. doi: 10.1016/j.ajog.2015.08.040 26428501 PMC4774647

[7] Ocviyanti D, Wahono WT. Risk Factors for Neonatal Sepsis in Pregnant Women with Premature Rupture of the Membrane. J Pregnancy. 2018 Oct 1;2018:4823404. doi: 10.1155/2018/4823404 30402288 PMC6191960

[8] Higgins RD, Saade G, Polin RA, Grobman WA, Buhimschi IA, Watterberg K, et al. Evaluation and Management of Women and Newborns With a Maternal Diagnosis of Chorioamnionitis: Summary of a Workshop. Obstet Gynecol. 2016 Mar;127:426–436. doi: 10.1097/AOG.0000000000001246 26855098 PMC4764452

[9] Hannah ME, Ohlsson A, Farine D, Hewson SA, Hodnett ED, Myhr TL, et al. Induction of labor compared with expectant management for prelabor rupture of the membranes at term. TERMPROM Study Group. N Engl J Med. 1996 Apr 18;334:1005–10. doi: 10.1056/NEJM199604183341601 8598837

[10] SIGO. Linee guida, raccomandazioni. Induzione al travaglio di parto.2016.

[11] Saccone G, Berghella V. Antibiotic prophylaxis for term or near-term premature rupture of membranes: metaanalysis of randomized trials. Am J Obstet Gynecol. 2015 May;212:627.e1–9. doi: 10.1016/j.ajog.2014.12.034 25555659

[12] Prevention of Group B Streptococcal Early-Onset Disease in Newborns: ACOG Committee Opinion, Number 797. Obstet Gynecol. 2020 Feb;135:e51–e72 Erratum in: Obstet Gynecol. 2020 Apr;135:978–979. doi: 10.1097/AOG.0000000000003668 31977795

[13] Polin RA, and the Committee on Fetus and Newborn. Management of neonates with suspected or proven early-onset sepsis. Pediatrics 2012;129:1006–15. doi: 10.1542/peds.2012-0541 22547779

[14] Benitz WE, Wynn JL, Polin RA. Reappraisal of guidelines for management of neonates with suspected early-onset sepsis. J Pediatr 2015;166:1070–4. doi: 10.1016/j.jpeds.2014.12.023 25641240 PMC4767008

[15] Cotten CM. Antibiotic stewardship: reassessment of guidelines for management of neonatal sepsis. Clin Perinatol 2015;42:195–206. doi: 10.1016/j.clp.2014.10.007 25678005 PMC4888789

[16] Verani JR, McGee L, Schrag SJ. Division of Bacterial Diseases, National Center for Immunization and Respiratory Diseases, Centers for Disease Control and Prevention (CDC). Prevention of perinatal group B streptococcal disease—revised guidelines from CDC, 2010. MMWR Recomm Rep. 2010 Nov 19;59:1–36. 21088663

[17] Middleton P, Shepherd E, Flenady V, McBain RD, Crowther CA. Planned early birth versus expectant management (waiting) for prelabour rupture of membranes at term (37 weeks or more). Cochrane Database Syst Rev. 2017 Issue 1. 10.1002/14651858.CD005302.pub3 PMC6464808 28050900

[18] ACOG Practice Bulletin No. 106: Intrapartum fetal heart rate monitoring: nomenclature, interpretation, and general management principles. American College of Obstetricians and Gynaecologists. Obstet. Gynecol. 2009 Jul;114:192–202. doi: 10.1097/AOG.0b013e3181aef106 19546798

[19] da Graça Krupa F, Cecatti JG, de Castro Surita FG, Milanez HM, Parpinelli MA. Misoprostol versus expectant management in premature rupture of membranes at term. BJOG. 2005 Sep;112:1284–90. doi: 10.1111/j.1471-0528.2005.00700.x 16101609

[20] Maqbool S, Usmani AS, Bano B. Comparison of induction and expectant management of prelabour rupture of membranes at term for maternal outcome. Pakistan Journal.

[21] Braunholtz DA, Edwards SJL, Lilford RJ. Are Randomized Clinical Trials Good for Us (in the Short Term)? Evidence for a ‘Trial Effect. 2001 Journal of Clinical Epidemiology 54:217–24. doi: 10.1016/s0895-4356(00)00305-x 11223318

[22] Term Prelabour Rupture of Membranes (Term PROM). RANZCOG guidelines, March 2020.

